# Low temperature stress and changes in the lignin content and composition in *Eucalyptus globulus*

**DOI:** 10.1186/1753-6561-5-S7-P102

**Published:** 2011-09-13

**Authors:** Jullyana Moura-Sobczak, Uiara Souza, Eduardo Kiyota, Paulo Mazzafera

**Affiliations:** 1 1. Departamento de Fisiologia Vegetal. Instituto de Biologia - Unicamp. CEP: 13083-970 Campinas, SP, Brazil; 2 4. Departamento de Fisiologia Vegetal. Instituto de Biologia - Unicamp. CEP: 13083-970 Campinas, SP, Brazil

## 

It is known that many abiotic stresses, such as mineral deficiency, drought, UV-B radiation, wind and low temperatures, alter the quantity and composition of lignin in several species [1]. The aim of this work was to verify if the cold stress may cause changes in the quantity and composition of lignin in *E. globulus*. Additionally, we analyzed whether these changes can also be beneficial from the industrial point of view, that is, if this would allow a better extractability of lignin in this species, which is a desirable feature in the manufacture of paper. During the day, all plants were kept in a greenhouse at room temperature but at night, half of plants was transferred to growth chamber at 12 °C and half to 25 °C. These conditions were kept during 20 days. The stems of plants from the two groups were collected and analyzed for total lignin with thioglycolic acid [2] and submitted to thioacidolysis and GC-MS analysis for the determination of the monomeric lignin composition [3]. Part of the material was used to determine the digestibility of cellulose [4].

It was observed that cold reduced the accumulation of lignin in *E. globulus*. GC-MS analysis showed that the proportion of the S/G was reduced in plants subjected to low temperatures and it was also observed a lower digestibility of cellulose in these plants, indicating that this lignin could be more difficult to be removed in industrial processes of papermaking.

These results have been related with gene expression studies for the enzymes of the lignin biosynthesis pathway and may contribute to understand the processes controlling lignin deposition in eucalyptus.

## References

[B1] Moura JCMS Bonine CAV Viana JOF Dornelas MC Mazzafera P Abiotic and Biotic Stresses and Changes in the Lignin Content and Composition in Plants J Integr Plant Biol 2010 52 360 376 10.1111/j.1744-7909.2010.00892.x 20377698

[B2] Lange BM Lapierre C Sandermann HJR Elicitor-induced spruce stress lignin Plant Physiol 1995 108 1277 1287 12228544 10.1104/pp.108.3.1277 PMC157483

[B3] Rolando C Monties B Lapierre C Lin S, Dence C Thioacidolysis Methods in Lignin Chemistry 1992 Berlin: Springer-Verlag 334 349

[B4] Chen F Dixon RA Lignin modification improves fermentable sugar yields for biofuel production Nat Biotechnol 2007 25 759 761 10.1038/nbt1316 17572667

